# Nanoscale magnesium hydride alleviates hypoxia-induced myelination deficits in zebrafish

**DOI:** 10.4103/mgr.MEDGASRES-D-25-00104

**Published:** 2026-01-23

**Authors:** Jia-Lin Li, Qiang Chen, Zheng-Hao Li, Dao-Jie Xu, Cheng He, Peng Liu

**Affiliations:** 1 Institute of Neuroscience and Key Laboratory of Molecular Neurobiology of Ministry of Education, Naval Medical University, Shanghai, China; 2 Department of Diving Medicine, Navy Medical Center, Naval Medical University, Shanghai, China; 3 Department of Anesthesiology, Huashan Hospital, Fudan University, Shanghai, China

**Keywords:** hydrogen, hypoxia, mitochondrial membrane potential (MMP), myelination, nanoscale magnesium hydride (MgH_2_), oligodendrocyte progenitor cells (OPCs), oligodendrocyte, oxidative stress, reactive oxygen species (ROS), zebrafish

## Abstract

Defects in myelination impair nerve impulse conduction and functional connectivity, which could lead to cognitive, behavioral and motor deficits in various neurological disorders. Adequate oxygen delivery is vital for brain development, while hypoxia in newborns tends to result in developmental deficiencies in myelination of the brain. The disruption of oligodendrocytes and their progenitor cells caused by hypoxia has been well researched. Nonetheless, the impairing dynamic myelination process is still unclear. Utilizing zebrafish as a model, we established hypoxia via cobalt chloride exposure or low oxygen (8%) incubation. Hypoxia significantly reduced oligodendrocyte progenitor cell numbers in the dorsal spinal cord, impaired migration velocity and suppressed proliferation. Myelination deficits were evident through decreased myelin sheath segment intensity in Tg(MBP:eGFP-CAAX) larvae. Time-lapse imaging revealed compromised dynamic myelination by individual oligodendrocytes under hypoxia, with fewer sheaths and reduced extension rates. Mechanistically, hypoxia elevated reactive oxygen species levels and disrupted mitochondrial membrane potential in cultured rat oligodendrocyte progenitor cells. Nanoscale magnesium hydride, a hydrogen-releasing agent, attenuated these effects. *In vivo*, magnesium hydride treatment rescued oligodendrocyte progenitor cell numbers and enhanced myelinogenesis capacity in hypoxic zebrafish. These findings demonstrate that magnesium hydride mitigates hypoxia-induced oxidative stress and mitochondrial dysfunction, thereby alleviating myelination deficits.

## Graphical abstract

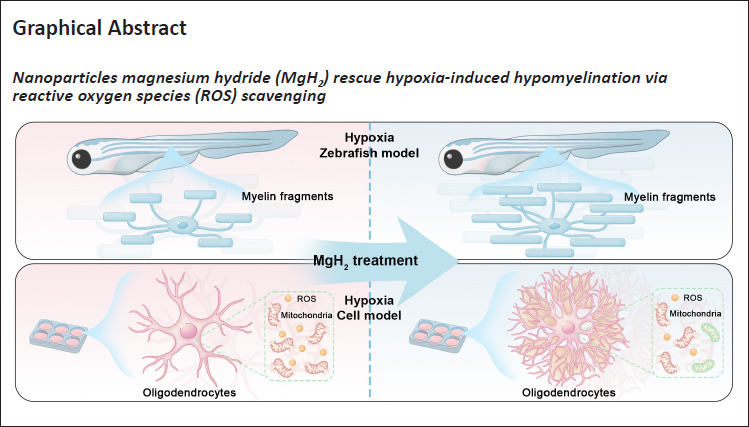

## Introduction

Sufficient oxygen delivery is essential for proper brain development, particularly during the critical early stages of life. In newborns, oxygen deprivation, or hypoxia, often leads to developmental abnormalities in the white matter.^1^ Under prolonged hypoxic-ischemic stress, oligodendrocyte progenitor cells (OPCs) undergo cell death, resulting in defective myelination, which triggers neurological dysfunction, disability, and mortality.^2^,^3^,^4^ Although much is known about the disruption in functioning of OPCs or oligodendrocytes (OLs) by hypoxia *in vitro* and *in vivo*,^5^,^6^,^7^ how dynamic myelination is impaired under hypoxia condition is still unclear.^8^,^9^

Zebrafish has emerged as a powerful model to visualize the dynamic myelinating procession of OLs *in vivo*.^10^,^11^ Due to its transparent larval development, zebrafish facilitates live imaging studies, enabling dynamic visualizations of cellular and molecular processes of OLs in real time studies.^11^,^12^,^13^ Furthermore, Zebrafish share significant genetic and physiological similarities with mammals, including humans, making them a relevant model for studying complex biological processes like myelination.^10^,^14^ Zebrafish genome is well-characterized and highly amenable to genetic manipulation, including the use of clustered regularly interspaced short palindromic repeats (CRISPR)/CRISPR-associated protein 9 for targeted mutagenesis.^10^,^12^ Thus, zebrafish provides a useful model for investigating the function and underlying mechanism of hypoxia on dynamic myelination *in vivo.*

Hydrogen, a novel anti-oxidant, is cell membrane-permeable and can target organelles. It selectively scavenges highly reactive species like hydroxyl radical (·OH) and peroxynitrite- (ONOO-) without disrupting essential metabolic oxidation,^15^ making it a promising therapeutic agent for hypoxia. Ohsawa et al.^16^ have reported that hydrogen ameliorated focal ischemic by buffering the effects of oxidative stress. Hydrogen-rich saline could restore behavioral deficits following hypoxia-ischemic in neonatal mice.^17^ In addition, hydrogen could also alleviate hypoxia-induced kidney and lung injury.^18^,^19^ Nanoscale magnesium hydride (MgH_2_) is an efficient magnesium-based hydrogen storage material that can sustainably produce massive hydrogen through the following reaction: MgH_2_ + 2H_2_O = Mg(OH)_2_ + 2H_2_.^20^,^21^ However, its efficacy in alleviating hypoxia-induced OLs damage remains underexplored.

In this study, we characterized the dynamic changes in OLs and myelination in a zebrafish hypoxic model. Additionally, we explored the therapeutic potential of nanoscale MgH_2_ as a novel intervention for hypoxia-induced myelination defects.

## Methods

### Zebrafish husbandry

Adult zebrafish (Danio rerio, China Zebrafish Resource Center, license No. CZRC-CZ1/CZ3) were maintained in a dedicated aquaculture facility at 28.5°C with a 14-hour light/10-hour dark cycle. They were provided with brine shrimp twice per day. Freshly fertilized eggs were produced by pairwise mating and raised in dish containing system water. All zebrafish experiments were approved by the Institutional Animal Care and Use Committees of Naval Medical University (approval No. 20230310-017, on March 10, 2023) and carried out according to the Animal Research Reporting of *in vivo* Experiments (ARRIVE) guidelines^22^ and to relevant guidelines and regulations.

The zebrafish lines used in this study included: wild-type AB line, Tg(MBP:eGFP-CAAX): Membrane eGFP (eGFP-CAAX) in MBP^+^ cells (OLs); Tg(MBP:Gal4) & Tg(5×UAS:GFP): Gal4 specifically expressed in MBP^+^ cells (OLs); Tg(Olig2:eGFP): eGFP in Olig2^+^ cells (OPCs and OLs); Tg(Sox10:mRFP): Membrane RFP (mRFP) in Sox10^+^ cells (OPCs and OLs). The Tg(Olig2:eGFP) transgenic line and the Sox10:mRFP plasmid were provided by University of Colorado School of Medicine. Tg(5×UAS:GFP) are kindly provided by Center for Excellence in Brain Science and Intelligence Technology, Chinese Academy of Sciences. The transgenic lines Tg(Sox10:mRFP), Tg(MBP:Gal4) and Tg(MBP:eGFP-CAAX) were created by injecting plasmid Sox10:mRFP, MBP:Gal4, MBP:eGFP-CAAX, together with I-SceI enzyme, into one-cell embryos.

### Hypoxia treatment

Hypoxia-like-state was established by cobalt chloride (CoCl_2_) application as previously described.^23^,^24^,^25^ Zebrafish embryos were dechorionated manually at 24 hours post-fertilization (hpf) and exposed to a range of concentrations of CoCl_2_ (0, 1, 2.5, 5, 10, and 20 mM). Subsequently, the concentration of CoCl_2_ for our subsequent experiments was determined to be 2.5 mM by morphological analysis of larval zebrafish. Larvae were incubated in CoCl_2_ before imaging at designated time points.

True hypoxic condition which was achieved by incubating larvae in a sealed box with 8% oxygen concentration (HERACELL VIOS 160i, Thermos Scientific, Waltham, MA, USA) from 1 day post-fertilization (dpf), which was similar as previous reported.^24^,^25^

### Quantitative polymerase chain reaction

Zebrafish were anesthetized with tricaine (160 mg/L; MS222, Sigma-Aldrich, St. Louis, MO, USA) by immersion until cessation of opercular movement (~1–3 minutes). The larvae were transferred to fresh system water for recovery. Zebrafish larvae were placed in tricaine (500 mg/L) solution for 10–30 minutes until death was confirmed by cessation of opercular (gill) movement. Each sample RNA was reverse-transcribed into complementary DNA with Evo M-MLV RT Master Mix (Accurate Biotechnology (Hunan) Co., Ltd., Changsha, China). Quantitative polymerase chain reaction was performed on a LightCycler 96 apparatus (Roche) using the SYBR Green Pro Taq HS Premix (Accurate Biotechnology (Hunan) Co., Ltd.). Gene expression was expressed as the mRNA level, which was normalized to that of a standard housekeeping gene (β-actin) using the ΔΔCT method.^26^ The primer pairs were as follows: for hypoxia inducible factor 1-alpha a (*hif1αa*), F: 5’-CTC AGC CGC CAC ACT TTA GA-3’; R: 5’-GCC CCT TCA CAA AAA GGC TG-3’; for *hif1αb*, F: 5’-CGC AGG AAG GAG AAG TCC AG-3’; R: 5’-GAG CTG GTG TGC TAA CTC GT-3’; for β*-actin*, F: 5’-TCC ATT GTT GGA CGA CCC AG-3’; R: 5’-TGG GCC TCA TCT CCC ACA TA-3’.

### Western blot analysis

Zebrafish larvae were homogenized in radioimmunoprecipitation assay buffer that was supplemented with protease inhibitor cocktail (Beyotime, Shanghai, China). Larvae lysates were subjected to Western blot analysis using rabbit anti-Hif1α monoclonal antibody (1:500, Cell Signaling Technology, Danvers, MA, USA, Cat# 36169, RRID: AB_2799095) and mouse anti-β-actin monoclonal antibody (1:2000, Proteintech, Wuhan, China, Cat# 66009, RRID: AB_2919581) overnight at 4°C, followed by incubation with 800CW-labeled anti-rabbit IgG (1:1000, LI-COR Biosciences, Lincoln, NE, USA, Cat# 926-32213, RRID: AB_2715510) and 680RD-labeled anti-mouse IgG secondary antibody (1:1000, LI-COR Biosciences, Cat# 926-68072, RRID: AB_2814912) for 2 hours at room temperature. The protein bands were analyzed and quantified using Image Lab (ODYSSEY CLX, LI-COR, Lincoln, NE, USA), normalizing target proteins to β-actin bands.

### Transgenic lines and time-lapse imaging

The study utilized transgenic zebrafish lines expressing red/green fluorescent protein (RFP/GFP) under control of OLs-specific promoters to visualize OPCs and myelination. Time-lapse imaging was performed using a confocal microscope (Dragonfly 200, ANDOR, Belfast, Northern Ireland, UK) with lenses (CFI Plan Apochromat 20X, 0.75, Nikon, Tokyo, Japan) from 3 to 5 dpf, and quantified using ImageJ software (1.53t, NIH, Bethesda, MD, USA).^27^ Zebrafish were anesthetized with tricaine (160 mg/L) and mounted laterally in 2% low-melting-point agarose on glass-bottom dishes. The temperature throughout imaging was monitored at 26–28.5°C. Larvae were checked for good blood circulation and general health prior to imaging. Images were captured sequentially at 24-hour intervals. Between imaging intervals, the zebrafish embryos were dissected carefully from the low-melting-point agarose and transferred to a fresh system of water to ensure sustained physiological viability throughout extended experiments (3–5 dpf). After imaging, zebrafish larvae were placed in tricaine (500 mg/L) solution for 10–30 minutes until death was confirmed by cessation of opercular (gill) movement.

In the figures, all zebrafish images are shown in lateral view, anterior to the left and dorsal on top. For morphology analyses of brightfield images, we used Fiji/ImageJ to mark the rostral and caudal ends of each larva (mouth and tail fin) to measure body length. For consistency, bright field and fluorescent images were finally taken from comparable regions of the spinal cord. Fluorescent images were acquired as z-stacks (z-step, 2 µm) and a resolution of 5000 × 5000 pixels. The images taken for cell counting analyses were evaluated using the Fiji (ImageJ) Cell counter plugin (Kurt De Vos, University of Sheffield). The images taken for fluorescence intensity measurements were acquired with identical conditions and mean fluorescence intensity from the stitched maximum intensity z-projections was obtained for each fish by using the measurement function of Fiji. For the measurement of the SRY-box transcription factor 10-positive (Sox10^+^) and myelin basic protein-positive (MBP^+^) area, the “wand” tool (with a constant threshold set manually with the first 2–3 samples and used throughout the dataset) was used to select the regions of interest while blinded to experimental condition, and the fluorescence intensity was used to quantify the result of area.

### Quantification of oligodendrocyte progenitor cell dynamics and myelination

ImageJ software was employed to quantify OPC number, migration, proliferation, and dorsal/ventral myelination. Fluorescent intensity and area were measured to assess myelination, while cell counts and tracking were used to evaluate OPC dynamics. OPC-migration velocity was calculated by dividing the distance traveled by the total traveling time that excluded the periods when they were stationary. To reduce the bias and collect more information, the data from eight somites were counted.

### Single oligodendrocyte labeling

To mosaically label OLs, fertilized eggs were microinjected as previously described^28^ at the one-cell stage with 1 nL of solution containing 25 ng/μL Sox10:eGFP-CAAX plasmid DNA and I-SceI. Animals were screened at 3 dpf for isolated OL.

### Primary oligodendrocyte progenitor cell culture

As described previously,^29^ primary OPCs were isolated from postnatal day 0 (P0) Sprague-Dawley rats (Jihui Laboratory Animal Care Co. Ltd., Shanghai, China; license No. SCXK (Hu) 2022-0009). Rat pups were placed on ice for anesthetization and killed by decapitation. Mixed cortical glial cultures were prepared from P0 rat pups and maintained in Dulbecco’s Modified Eagle Medium (Invitrogen, Carlsbad, CA, USA) containing 10% fetal bovine serum (Gibco, Grand Island, NY, USA) for 10 days at 37°C in a 5% CO_2_ atmosphere. Following this incubation, the cultures underwent mechanical separation through sequential shaking at 180 r/min for 1 hour and subsequently at 200 r/min for 18 hours with fresh medium replacement by using the digital shaking incubator (WS-600R, WIGGENS, Beijing, China). The liberated OPCs were collected and subjected to a differential adhesion step in uncoated Petri dishes to remove contaminating cell types. Purified OPCs were then seeded onto poly-L-lysine-coated culture vessels (Sigma-Aldrich) and maintained in proliferation medium containing 0.1% platelet-derived growth factor-AA (Peprotech, Rocky Hill, NJ, USA), 2% B27 supplement (Gibco), 1% N_2_ supplement (Gibco), and 0.1% basic fibroblast growth factor (Peprotech). For OL maturation studies, OPCs were transferred to Neurobasal medium supplemented with 2% B27 (Gibco).

### Immunofluorescence staining

Primary rat OLs were fixed, permeabilized, and incubated with primary rat monoclonal anti-MBP antibody (1:50, Abcam, Cambridge, MA, USA, Cat# ab7349, RRID: AB_305869) overnight at 4°C, followed by incubation with TRITC-conjugated donkey anti-rat secondary antibody (1:200, Jackson ImmunoResearch, West Grove, PA, USA) and counterstained with Hoechst33342 (1:1000, Sigma-Aldrich) for 2 hours at room temperature. Fluorescence images were captured using a fluorescence microscope (Dragonfly 200, ANDOR).

### Measurement of reactive oxygen species generation

This method has been described previously.^30^ Reactive oxygen species assay kit (Beyotime) was used to detect the intracellular generation of reactive oxygen species (ROS). The cultured rat OLs were incubated in serum-free media with 10 μM 2,7-dichlorodihydrofluorescein diacetates at 37°C for 20 minutes. Next, the cells were washed three times with a serum-free medium. After the corresponding treatment, the cells were visualized by fluorescence microscopy (N1-E, Nikon).

### Mitochondrial membrane potential measurement

Mitochondrial membrane potential (mitochondrial membrane potential) was assessed using a JC-1-based mitochondrial membrane potential assay kit (MCE, Monmouth Junction, NJ, USA, HY-K0601) following the manufacturer’s protocol. Briefly, the cultured rat OLs were collected, washed once with ice-cold PBS, and resuspended in a mixture of 200 μL culture medium and 1 μL JC-1 staining solution. The suspension was incubated in the dark at 37°C for 20 minutes. After incubation, cells were washed three times with ice-cold staining buffer. The cells were visualized by fluorescence microscopy.

### Administration of nanoscale magnesium hydride

Nanoscale MgH_2_ powder (provided by the Center of Hydrogen Science, Shanghai Jiao Tong University) was suspended in propylene glycol, and for cell and animal experiments.

For the rat cell experiment, the CoCl_2_ + MgH_2_ and CoCl_2_ groups received 100 μM MgH_2_ or an identical dose of propylene glycol on the first day of OPC differentiation.

For the zebrafish study, embryos were mechanically dechorionated at 1 dpf and the larvae were randomly divided into CoCl_2_ + MgH_2_ and CoCl_2_ groups. They were then transferred into small petri dishes containing 100 μM MgH_2_ or an identical dose of propylene glycol. The solutions were renewed every day during the imaging period.

### Statistical analysis

All graphs and statistical tests were carried out using GraphPad Prism (Version 9.5.1, GraphPad Software, Boston, MA, USA, www.graphpad.com). Data are presented as mean ± standard error of mean (SEM). Data distribution was tested normally. Differences between treatment and control groups were analyzed using a two-tailed unpaired/paired Student’s *t*-test. Two-way analysis of variance test was performed by multiple comparisons or pairwise comparisons followed by Bonferroni *post hoc* test. A *P*-value of less than 0.05 was considered statistically significant.

## Results

### Hypoxia causes developmental defects in oligodendrocyte cells

**Figure 1:**
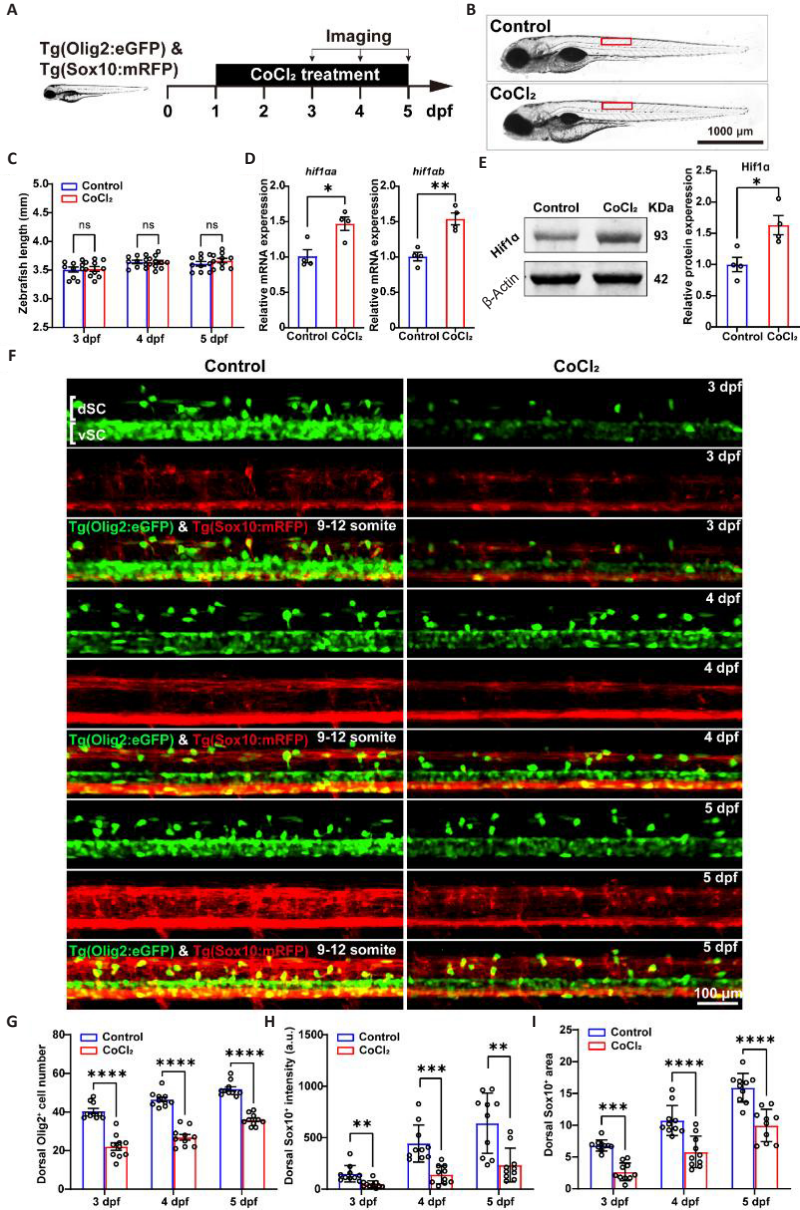
Developmental deficiency of oligodendroglial lineage cells by CoCl_2_-induced hypoxia-like state. (A) Experimental timeline: From the dechorionation of zebrafish embryos at 1 dpf to the CoCl_2_ treatment starting at 1 dpf, followed by imaging at 3, 4, and 5 dpf. Created with Adobe Photoshop (Version 20.0.0). (B) Bright field imaging shows the normal morphology and the area (9–12 somites as shown in the red box) being analyzed in F. (C) Measurements of larval length during development (*P* = 0.995 (3 dpf), 1.000 (4 dpf), 0.690 (5 dpf), *n* = 10 larvae). (D) qPCR analyses of zebrafish larval *hif1αa* and *hif1αb* mRNA expression after 48 hours of CoCl_2_ treatment. (E) Western blot analysis of larval Hif1α protein expression after 48 hours of CoCl_2_ treatment. (F) The fluorescent images of Tg(Olig2:eGFP) and Tg(Sox10:mRFP) transgenic lines by CoCl_2_ treatment and controls. CoCl_2_ treatment reduced oligodendroglial lineage cell numbers and myelin sheath segments from 3 dpf to 5 dpf. Scale bar: 100 μm. (G) Quantification of dorsal Olig2^+^ oligodendroglial lineage cell numbers (*P* < 0.001 (3 dpf), < 0.001 (4 dpf), < 0.001 (5 dpf), *n* = 10 larvae). (H, I) Quantification of dorsal Sox10^+^ area (*P* < 0.0001 (3 dpf), = 0.0002 (4 dpf), < 0.0001 (5 dpf), *n* = 10 larvae) and intensity (*P* = 0.0001 (4 dpf), = 0.0012 (5 dpf), *n* = 10 larvae). Two-way analysis of variance followed by Bonferroni *post hoc* test (C, G–I) and two-tailed paired *t*-test (D, E) were used. *P < 0.05, **P < 0.01, ***P < 0.001, ****P < 0.0001; error bars indicate mean ± SEM. CoCl_2_: Cobalt chloride; dpf: day(s) post-fertilization; Hif1α: hypoxia inducible factor-1α; ns: not significant; Olig2: oligodendrocyte transcription factor 2; qPCR: quantitative real-time polymerase chain reaction; SEM: standard error of mean; Sox10: SRY-box transcription factor 10.

To establish CoCl_2_-induced hypoxia model in zebrafish embryos, we screened a range of concentrations of CoCl_2_ (0, 1, 2.5, 5, 10, and 20 mM) to examine the hypoxia stress and developmental conditions (**Additional Figure 1A** and **B**). We applied CoCl_2_ from 1 dpf to 5 dpf and checked the morphological phenotypes at 3–5 dpf, when the embryos underwent extensive myelination in zebrafish central nervous system (**Figure 1A**). We found that, the death rate and the developmental defects, such as edema malformation, increased significantly when the concentration was higher than 5 mM at 3 dpf (**Additional Figure 1B** and **C**). However, when it was 2.5 mM and below, the morphological phenotypes of embryos under hypoxia stress (**Figure 1B**), such as the total length (**Figure 1C**), eye area (**Additional Figure 2A**), and body area (**Additional Figure 2B**), were comparable with controls. To confirm the hypoxic-like state for larval zebrafish by CoCl_2_ treatment, we examined the expression level of Hif-1α, a regulator of hypoxia-induced transcription, at 3 dpf. Because of whole-genome duplication events during evolution, there are two copies of *hif1α*, *hif1aa* and *hif1ab*, in zebrafish and other teleost. The results showed that Hif1α expression increased significantly in response to CoCl_2_ application both at mRNA (**Figure 1D**) and protein levels (**Figure 1E**). Thus, in the following studies, 2.5 mM CoCl_2_ was used to induce hypoxia-like stress in zebrafish embryos during developmental myelinating stage.

Moreover, we examined the myelinating phenotypes in the spinal cord of transgenic lines Tg(Olig2:eGFP), in which oligodendroglial lineage cells were labeled by GFP, and Tg(Sox10:mRFP), which marked the myelin sheaths by mRFP, at 3–5 dpf with intervals of 24 hours (**Figure 1F**). We found that, compared with controls, CoCl_2_ treatment significantly decreased the number of oligodendroglial lineage cells in the dorsal spinal cord, which were originated and migrated from the ventral region, from 3 dpf (the stage of myelination initiation of the spinal cord) to 5 dpf (the stage of peak myelination of early embryo development), indicating fewer OPCs/OLs in dorsal spinal cord under hypoxic conditions (**Figure 1G**), consistent with a previous study.^25^ Moreover, the area and intensity of mRFP, which labeled the myelin sheath segments, were also reduced dramatically in the dorsal spinal cord from 3 to 5 dpf with CoCl_2_ treatment (**Figure 1H** and **I**).

**Figure 2:**
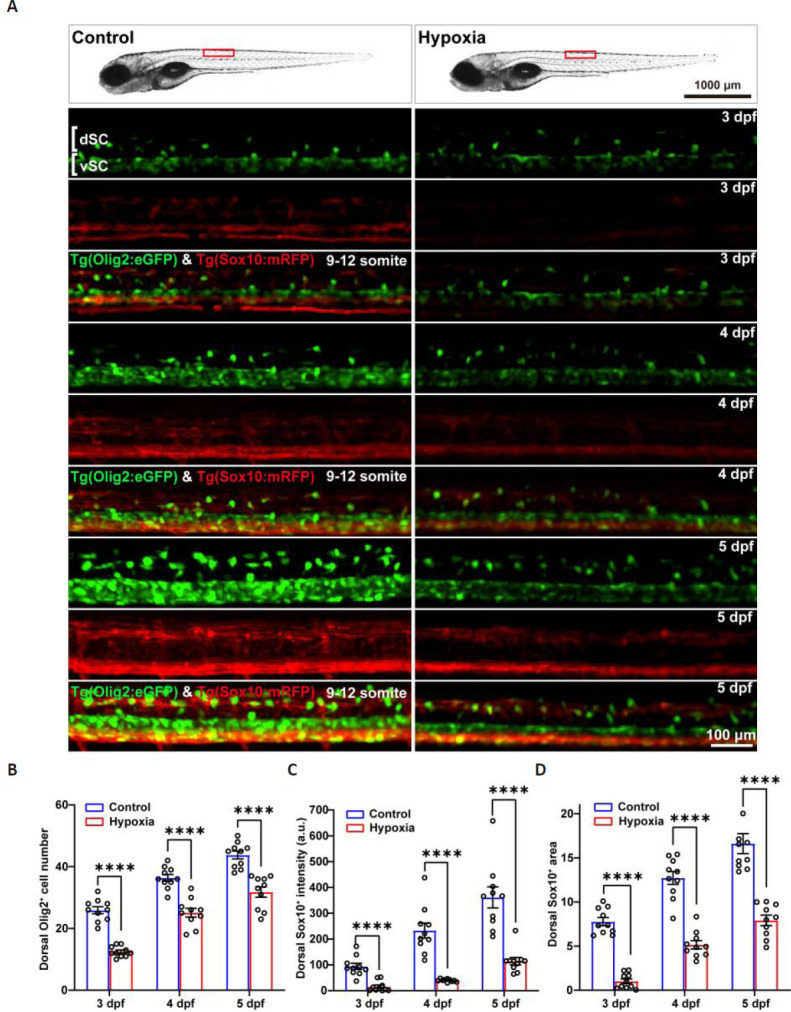
Developmental deficiency of oligodendroglial lineage cells under low oxygen concentration-induced hypoxia state. (A) The bright field and fluorescent images of Tg(Olig2:eGFP) and Tg(Sox10:mRFP) transgenic lines under hypoxic and control conditions. Low oxygen treatment reduced oligodendroglial lineage cell numbers and myelin sheath segments from 3 dpf to 5 dpf. Scale bar: 100 μm. (B) Quantification of dorsal Olig2^+^ oligodendroglial lineage cell numbers. (C, D) Quantification of dorsal Sox10^+^ area and intensity. Two-way analysis of variance followed by Bonferroni *post hoc* test (B–D) was used. ****P < 0.0001; error bars indicate mean ± SEM. dpf: Days post-fertilization; Olig2: oligodendrocyte transcription factor 2; SEM: standard error of mean; Sox10: SRY-box transcription factor 10.

In addition, we analyzed oligodendroglial development and the myelination pattern in the spinal cord of zebrafish in true hypoxic condition which was achieved by incubating larvae in a sealed box with 8% oxygen concentration. We found a severe defect in the development of oligodendroglial lineage cells under hypoxic conditions induced by low oxygen concentration, which was similar to the results with CoCl_2_ treatment (**Figure 2**). Thus, these results demonstrated that hypoxia impaired the developmental migration and myelination in zebrafish spinal cord.

### Hypoxic stress interferes with oligodendrocyte progenitor cell migration and proliferation

In order to further characterize the dynamic migration and proliferation of OPC in the spinal cord under CoCl_2_-induced hypoxia stress, we performed time-lapse imaging of the middle region along anterior-to-posterior axis of the spinal cord of Tg(Olig2:eGFP) transgenic line from 51 to 63 hpf at 2-hour intervals (**Additional Figure 3A**), when OPCs arose from the motoneuron progenitor domain of zebrafish ventral spinal cord and began to migrate to dorsal. We tracked the trajectory of OPC dorsal migration (**Additional Figure 3B**) and found that, compared with controls, the dorsal migrating OPC number was decreased significantly. Meanwhile, the OPC migration velocity was descended (**Additional Figure 3C** and **D**). Surprisingly, we also found that the number of proliferating OPCs (**Additional Figure 3E**), which divided from one into two daughter OPCs during the period of time-lapse imaging, reduced significantly under hypoxia stress. Together, these results indicated that CoCl_2_ treatment decreased OPCs’ mobility and proliferating rate in the spinal cord of developmental zebrafish larvae.

### Hypoxia leads to hypomyelination of oligodendrocytes

To further explore the function of CoCl_2_ treatment on myelination, we examined the number of mature OLs from 3 to 5 dpf with 24-hour intervals in the spinal cord of Tg(MBP:Gal4 & 5×UAS:GFP) transgenic line, which expressed GFP in the cytoplasm of mature OLs (**Additional Figure 4A**). The results showed that, compared with control, there was significant reduction of OL numbers both in the dorsal spinal cord and ventral spinal cord under hypoxia-like stress at 3–5 dpf, respectively (**Additional Figure 4B** and **C**). Moreover, the myelin sheath intensity, which was labeled by membrane-tethered GFP (GFP-CAAX) under the control of MBP promoter in Tg(MBP:eGFP-CAAX) transgenic line (**Additional Figure 4D**), was markedly decreased in the dorsal spinal cord and ventral spinal cord with CoCl_2_ treatment (**Additional Figure 4E** and **F**). Meanwhile, the myelin sheath segment area was also decreased in the dorsal spinal cord and ventral spinal cord in hypoxic-like environment (**Additional Figure 4G** and **H**). Furthermore, low oxygen concentration-induced hypoxia also induced severe hypomyelination in the dorsal spinal cord and ventral spinal cord of Tg(MBP:eGFP-CAAX) transgenic line from 3–5 dpf (**Additional Figure 5**). Taken together, these findings indicated the developmental hypomyelination in the spinal cord of zebrafish larvae by hypoxia stress.

### Hypoxia stress impairs dynamic myelination by individual oligodendrocytes

To further characterize the dynamic process of central nervous system myelination with CoCl_2_ treatment, we performed long-term time-lapse confocal imaging of the same OLs in the spinal cord of intact zebrafish larvae for larvae from 3 to 5 dpf with intervals of 24 hours (**Additional Figure 6A**). Individual OLs were mosaically labeled by GFP-CAAX under the control of OPCs/OLs-specific expressed Sox10 promoter (**Additional Figure 6B**). We found significant reductions in the number and length of myelin sheaths formed by individual OLs under hypoxia-like stress at 5 dpf (**Additional Figure 6C** and **D**). Furthermore, based on time-lapse imaging of individual OLs (**Additional Figure 6E**), we found that the extension of myelin sheaths, as revealed by the average length, the total length, and sheath number of myelin sheaths formed by individual OLs, was also inhibited with CoCl_2_ treatment from 3 to 5 dpf (**Additional Figure 6F–H**). Thus, hypoxia impairs the myelination of individual OLs in zebrafish spinal cord.

### Magnesium hydride improves the oligodendrocyte maturation defects and oxidative stress mediated by cobalt chloride-induced hypoxia *in vitro*

To further explore the roles of CoCl_2_-induced hypoxia on myelination, we designed *in vitro* OLs maturation experiments. We found that the percentage of MBP^+^ cells in cultured OLs was markedly decreased following CoCl_2_-treatment (**Additional Figure 7A** and **B**). This result indicates that CoCl_2_-induced hypoxia disrupted OLs maturation, consistent with the findings from zebrafish studies.

Additionally, we detected intracellular ROS levels in OLs using DCFH-DA after CoCl_2_-treatment. ROS generation was higher in the CoCl_2_-treated OPCs than the control group (**Additional Figure 7C** and **D**). Depolarization of mitochondrial membrane potential is a hallmark of mitochondrial dysfunction. While the CoCl_2_-treated OPCs showed strong green fluorescence indicating mitochondrial membrane potential depolarization, the controls emitted little green fluorescence, indicating normal relative (**Additional Figure 7E** and **F**).

**Figure 3:**
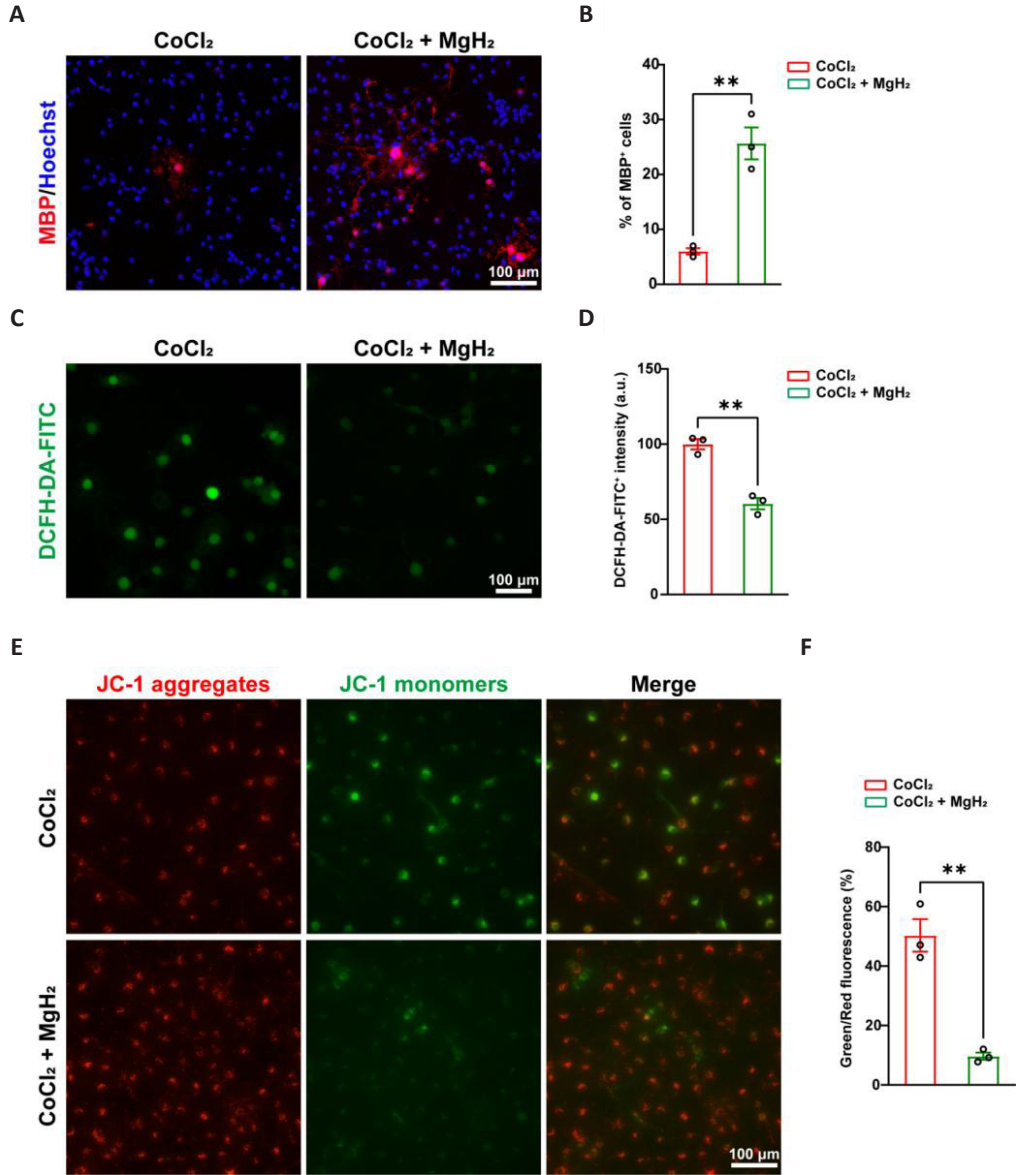
MgH_2_ improves the hypomyelination and oxidative stress mediated by CoCl_2_-induced hypoxia in vitro. (A) Immunostaining of MBP and Hoechst in the primary OLs in CoCl_2_ and CoCl_2_ + MgH_2_-treatment conditions. MgH_2_ treatment enhanced CoCl_2_-induced impairment of OPC differentiation. Scale bar: 100 μm. (B) Quantification of percentage of MBP^+^ cells. (C) Immunostaining of DCFH-DA-FITC in the primary OLs in CoCl_2_ and CoCl_2_ + MgH_2_-treatment conditions. MgH_2_ treatment reduced ROS levels of OLs after hypoxia. Scale bar: 100 μm. (D) Quantification of DCFH-DA-FITC intensity. (E) Immunostaining of JC-1 aggregates and monomers in the primary OLs in CoCl_2_ and CoCl_2_ + MgH_2_-treatment conditions. MgH_2_ treatment increased the MMP of OLs after hypoxia. Scale bar: 100 μm. (F) Quantification of percentage of JC-1 monomers/aggregates fluorescence. Two-tailed unpaired *t*-test (B, D, F) was used. **P < 0.01; error bars indicate mean ± SEM. CoCl_2_: Cobalt chloride; MBP: myelin basic protein; MgH_2_: magnesium hydride; MMP: mitochondrial membrane potential; OLs: oligodendrocytes; OPC: oligodendrocyte progenitor cell; ROS: reactive oxygen species; SEM: standard error of mean.

Nanoscale MgH_2_ is a new type of hydrogen storage material that can release large amounts of hydrogen. To explore the therapeutic effect of MgH_2_ on hypoxia-induced OL maturation defects *in vitro*, primary cultured OPCs were administered 100 μM MgH_2_. A significant increase in the proportion of MBP^+^ cells was observed after MgH_2_ treatment, compared with the CoCl_2_-treated group (**Figure 3A** and **B**). Meanwhile, MgH_2_ treatment could reduce the ROS levels of OPCs under CoCl_2_-induced hypoxic conditions (**Figure 3C** and **D**) and increase their mitochondrial membrane potential (**Figure 3E** and **F**). These results indicated that MgH_2_ treatment can affect OL maturation, suppress oligodendroglial oxidative stress, and protect mitochondrial function *in vitro.*

### Magnesium hydride improves the developmental deficiency of oligodendroglial lineage cells and hypomyelination by cobalt chloride-induced hypoxia in zebrafish

**Figure 4:**
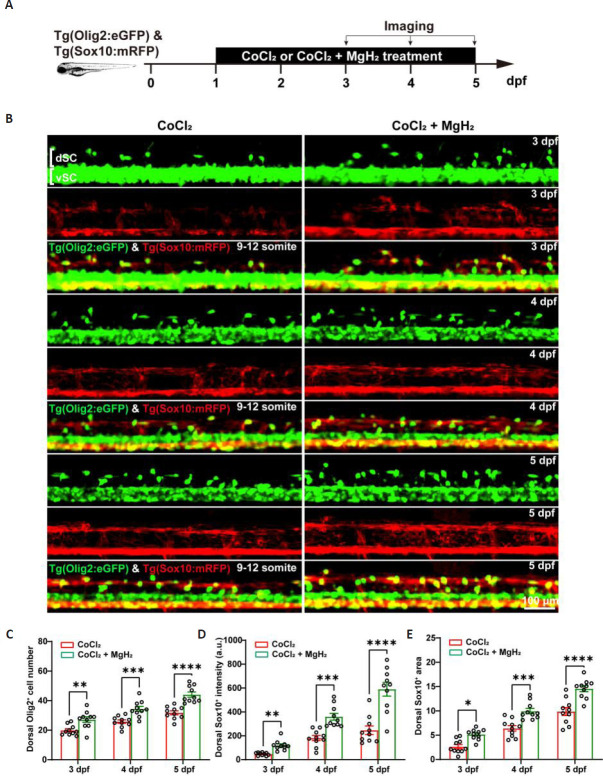
MgH_2_ improves the developmental deficiency of oligodendroglial lineage cells by CoCl_2_-induced hypoxia in zebrafish. (A) Experimental timeline: from the dechorionation of zebrafish embryos at 1 dpf to the CoCl_2_ or CoCl_2_ + MgH_2_-treatment starting at 1 dpf, followed by imaging at 3–5 dpf. Created with Adobe Photoshop (Version 20.0.0). (B) The fluorescent images of Tg(Olig2:eGFP) and Tg(Sox10:mRFP) transgenic lines by CoCl_2_ and CoCl_2_ + MgH_2_-treatment. MgH_2_ treatment increased oligodendroglial lineage cell numbers and myelin sheath segments from 3 dpf to 5 dpf after hypoxia. Scale bar: 100 μm. (C) Quantification of dorsal Olig^2+^ oligodendroglial lineage cell numbers (*P* = 0.0036 (3 dpf), = 0.0005 (4 dpf), < 0.0001 (5 dpf), *n* = 10 larvae). (D–F) Quantification of dorsal Sox10^+^ area (*P* = 0.0102 (3 dpf), = 0.0002 (4 dpf), < 0.0001 (5 dpf), *n* = 10 larvae) and intensity (*P* = 0.001 (3 dpf), < 0.0001 (4 dpf), < 0.0001 (5 dpf), *n* = 10 larvae). Two-way analysis of variance followed by Bonferroni *post hoc* test (C–F) was used. *P < 0.05, **P < 0.01, ***P < 0.001, ****P < 0.0001; error bars indicate mean ± SEM. CoCl_2_: Cobalt chloride; dpf: day(s) post-fertilization; MgH_2_: magnesium hydride; Olig_2_: oligodendrocyte transcription factor 2; SEM: standard error of mean; Sox10: SRY-box transcription factor 10.

To further explore the therapeutic effect of MgH_2_ *in vivo*, zebrafish were administered 100 μM MgH_2_ under CoCl_2_-induced hypoxia conditions. We examined the developmental phenotypes of oligodendroglial lineage cells in the spinal cord of Tg(Olig2:eGFP and Tg(Sox10:mRFP), from 3–5 dpf with intervals of 24 hours (**Figure 4A**). We found that, compared with the CoCl_2_-induced hypoxia group, CoCl_2_ treatment significantly increased the number of oligodendroglial lineage cells in the dorsal spinal cord from 3 to 5 dpf (**Figure 4B** and **C**). Moreover, the area and intensity of mRFP, which labeled the myelin sheath segments, were also induced dramatically in the dorsal spinal cord from 3 to 5 dpf with MgH_2_ treatment (**Figure 4D** and **E**). These results indicated that developmental defects in OLs under hypoxia are rescued by MgH_2_.

**Figure 5:**
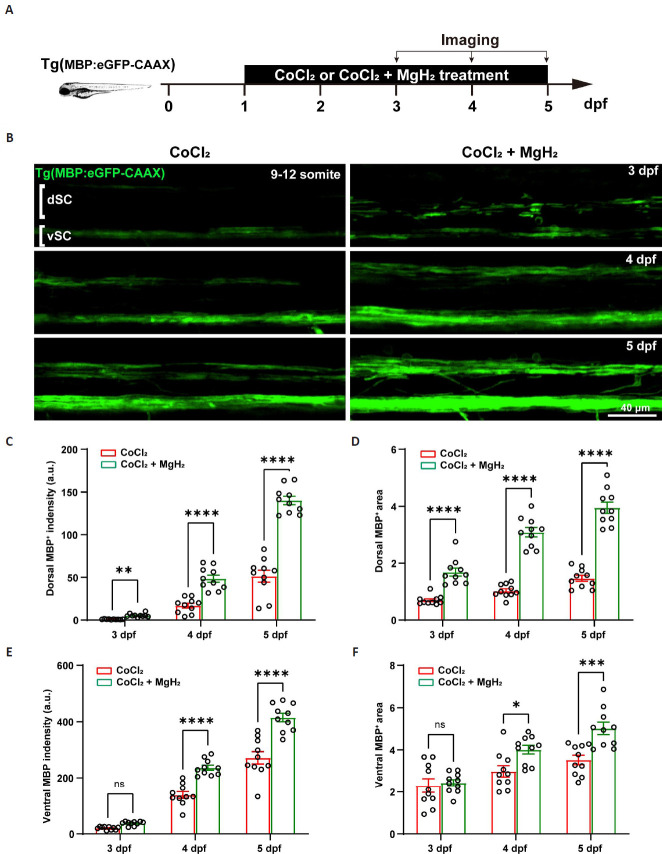
MgH_2_ improves the hypomyelination mediated by CoCl_2_-induced hypoxia in zebrafish. (A) Experimental timeline: from the dechorionation of zebrafish embryos at 1 dpf to the CoCl_2_ or CoCl_2_ + MgH_2_-treatment starting at 1 dpf, followed by imaging at 3–5 dpf. Created with Adobe Photoshop (Version 20.0.0). (B) Fluorescence imaging of myelination (9–12 somites). Longitudinal fluorescent images of zebrafish SC illustrate myelination levels. MgH_2_ treatment significantly increased the level of myelination in zebrafish from 3 dpf to 5 dpf after hypoxia. Scale bar: 100 μm. (C, D) Quantification of dorsal myelination. MBP^+^ intensity (*P* = 0.02 (3 dpf), < 0.0001 (4 dpf), < 0.0001 (5 dpf), *n* = 10 larvae) and area (*P* < 0.0001 (3 dpf), < 0.0001 (4 dpf), < 0.0001 (5 dpf), *n* = 10 larvae) in the dSC, demonstrating a significant increase in the CoCl_2_ + MgH_2_ group across all time points. (E, F) Quantification of ventral myelination. MBP^+^ intensity (*P* < 0.0001 (4 dpf), < 0.0001 (5 dpf), *n* = 10 larvae) and area (*P* = 0.0165 (4 dpf), = 0.0003 (5 dpf), *n* = 10 larvae) in the vSC. Two-way analysis of variance followed by Bonferroni *post hoc* test (C–F) was used. *P < 0.05, **P < 0.01, ***P < 0.001, ****P < 0.0001; error bars indicate mean ± SEM. CoCl_2_: Cobalt chloride; dpf: day(s) post-fertilization; dSC: dorsal SC; MBP: myelin basic protein; MgH_2_: magnesium hydride; ns: not significant; SC: spinal cord; SEM: standard error of mean; vSC: ventral SC.

To explore the therapeutic effect of MgH_2_ treatment on myelination, we examined the myelin sheath segments from 3 to 5 dpf with 24-hour interval in the spinal cord of Tg(MBP:eGFP-CAAX) transgenic line (**Figure 5A**). The results showed that, compared with the CoCl_2_-induced hypoxia group, MgH_2_ treatment markedly increased the density and area of MBP^+^ in the dorsal spinal cord at 3–5 dpf (**Figure 5B–D**). Meanwhile, the density and area of the myelin sheath in the ventral spinal cord were also increased at 4 and 5 dpf (**Figure 5E** and **F**). In summary, MgH_2_ can effectively ameliorate dynamic myelin deficits caused by CoCl_2_-induced hypoxia.

## Discussion

The effects of hypoxia were generalized, and hypoxia impaired the development and function of neurons and glia in the central nervous system. Neonatal hypoxic-ischemic episodes are known to trigger excitotoxicity, oxidative stress, and inflammation, leading to neuronal dysfunction and long-term neurological impairments.^3^,^31^ In both mice and rats, hypoxia reduces the volume of the cerebral cortex and corpus callosum, and eventually leads to liberal ventriculomegaly.^32^,^33^ Hypoxia also has been shown to disrupt synaptic development and glia-neuron interactions.^34^ In zebrafish, hypoxia caused a decrease in the number of synapses from the descending dopaminergic diencephalospinal tract to spinal cord motor neurons, resulting in decreased spontaneous swimming behavior in larva, and this motor impairment persisted into adulthood.^35^ Moreover, only 16-hour hypoxia from 5 dpf significantly decreased forebrain neural proliferation by 55%, and reduced the expression of Neurod1, glial fibrillary acidic protein and MBP, markers of determined neurons, glia and OLs, respectively.^36^ In addition, developmental hypoxic injury disrupts the pathfinding of forebrain neurons in zebrafish, leading to errors in which commissural axons fail to cross the midline.^37^ Here, we particularly focus on OL development and myelinogenesis in the spinal cord of zebrafish. Our results align with previous findings from zebrafish and rodent models, where hypoxia was both shown to induce irreversible deficits in myelination. Kanaan et al.^38^ demonstrated that chronic hypoxia leads to a persistent reduction in myelination in the corpus callosum despite the reversal of hypoxia-induced angiogenesis upon reoxygenation. Our finding also showed that hypoxia disrupted different developmental stages of oligodendroglial lineage cells, such as OPC migration, proliferation and OL myelination. Moreover, by single OL labeling, we showed that fewer myelin sheathes were generated under hypoxia, indicating that hypoxia severely disrupted myelinogenesis in zebrafish. Our data suggest the intervention to neonatal hypoxic-ischemia should focus on the dynamic developmental processes of OLs.

In the current study, we employed both CoCl_2_-induced chemical hypoxia and low-oxygen concentration exposure to establish two hypoxia models, enabling a comprehensive assessment of cellular responses of OLs under different hypoxic conditions. Notably, CoCl_2_-induced hypoxia serves as a reliable and controllable model that specifically targets the Hif-1α pathway, faithfully recapitulating key molecular features of hypoxic stress observed in mammalian systems. Similarly, we also detected the upregulation of *Hif-1α* gene and protein levels in zebrafish after CoCl_2_ treatment. We focused more exclusively on the CoCl_2_-induced model due to (1) the reproducibility and stability of CoCl_2_-mediated chemical hypoxia, (2) its widespread adoption in neuropathological studies for standardized comparison with prior literature,^25^ and (3) practical constraints in maintaining large-scale low-oxygen incubations during prolonged mechanistic and therapeutic studies. Importantly, our preliminary data confirmed that CoCl_2_ and physical hypoxia triggered comparable hypomyelination processes, validating the translational relevance of this approach for treatment.

The disruption of OPCs/OLs by hypoxia has been fully studied; however, the dynamic myelination under hypoxia is still unclear.^3^,^39^,^40^ Hypoxia has been shown to induce a wide range of cellular and molecular responses that compromise the integrity of the myelination process.^25^ Cerebral ischemia results in oxidative stress due to the generation of free radicals.^41^,^42^ Oxidative stress can induce cell death via DNA damage, lipid peroxidation, and change in protein structure and function.^6^,^40^,^41^,^43^ Activated microglia and astrocytes in releasing proinflammatory cytokines and increasing nitric oxide levels can also be detrimental to OPCs.^40^,^44^,^45^,^46^ Our findings are consistent with those, as we observed a decrease in OPCs and OLs numbers and a disruption in myelination pathways by using larval zebrafish. Notably, in an *in vitro* model of cellular hypoxia, there is an increase in ROS levels within the OLs during differentiation, accompanied by a depolarization of mitochondrial membrane potentials.

Ameliorating myelination defects under hypoxic conditions represents a promising therapeutic strategy for hypoxic-ischemic encephalopathy and cerebral ischemia.^3^,^47^,^48^ Our results indicate that reducing oxidative stress in OLs may effectively address this issue. In this study, nanoscale MgH_2_, an efficient magnesium-based hydrogen storage material, showed therapeutic potential in reducing oxidative stress and mitochondrial damage of OLs under hypoxia, which is consistent with our previous observations in microglia.^30^ In contrast, we previously found that nanoscale MgH_2_ did not exert a significant effect on OPC differentiation under physiological conditions,^30^ indicating that its therapeutic efficacy may be context-dependent and primarily effective in pathological settings. In general, hydrogen exerts several neuroprotective functions under hypoxic conditions. For example, it has been reported previously that hydrogen-rich saline promotes microglia M2 polarization and complement-mediated synapse loss to restore behavioral deficits following hypoxia-ischemia.^17^ Hydrogen has also been shown to protect against hypoxia/reoxygenation-induced neuron death by reducing oxidative stress.15 Moreover, hydrogen-rich water significantly attenuates hypoxia-ischemia-induced oxidative stress in brain pericytes partly through the nuclear factor erythroid 2-related factor 2/heme oxygenase-1 pathway.^49^ These findings highlight the potential of hydrogen-based therapies in the treatment of hypoxic injury in OLs and other neural cells.

Despite the promising results, there are a few limitations to this study worth mentioning. Although the CoCl_2_ chemical oxygen-deprived model mimics the myelin defects caused by hypoxia, it may not fully recapitulate the complexity of physiological hypoxia. Besides, the rat OPC hypoxia model provided mechanistic insights into anti-oxidative stress of nanoscale MgH_2_. However, whether nanoscale MgH_2_ also has non-targeted OL effects under hypoxic conditions, such as on vascular endothelial cells and microglia, requires in-depth study.

Our study reveals the dynamic process of myelinogenesis in living animal models under hypoxia stress. Our findings highlight the need for further research into the cellular mechanisms disrupted by hypoxia. Our study also indicates the therapeutic potential of nanoscale MgH_2_ to improve myelination defects under hypoxic conditions. Future research can harness the zebrafish hypoxia model as a powerful *in vivo* imaging platform to rapidly and efficiently screen a large number of anti-hypoxia candidates.

## Additional files:

***Additional Figure 1:*** *The morphology and survival probability of zebrafish larvae with CoCl_2_ treatment.*

Additional Figure 1 The morphology and survival probability of zebrafish larvae with CoCl_2_ treatment.
(A) Experimental timeline diagram of CoCl_2_ treatment. Created with Adobe Photoshop (Version 20.0.0) for production. (B) The survival probability of zebrafish larvae treated with different CoCl_2_ concentrations. The death rate and the developmental defects increased significantly when the CoCl_2_ concentration was higher than 5 mM at 3 dpf. (C) Dorsal view of zebrafish larvae with 5 mM CoCl_2_ treatment at 3 dpf. Scale bar: 1000 μm. Down: Zebrafish morphology with the treatment of 0, 1, 2.5, 5, 10, and 20 mM CoCl_2_ concentrations. Increasing concentrations of CoCl_2_ induced dose-dependent developmental toxicity in zebrafish, significantly elevating the incidence of body curvature, morphological deformities, and mortality. Scale bar: 200 μm. CoCl_2_: Cobalt chloride; dpf: day(s) post-fertilization.

***Additional Figure 2:*** *The eye and body area of zebrafish with 2.5 mM CoCl_2_ treatment.*

Additional Figure 2 The eye and body area of zebrafish with 2.5 mM CoCl_2_ treatment.
(A) The eye area of zebrafish larvae at 3–5 dpf in control and 2.5 mM CoCl_2_-treatment conditions. (B) The body area of zebrafish larvae at 3–5 dpf in control and 2.5 mM CoCl_2_-treatment conditions. Two-way analysis of variance followed by Bonferroni *post hoc* test (A, B), error bars indicate mean ± SEM. CoCl_2_: Cobalt chloride; dpf: days post-fertilization.

***Additional Figure 3:*** *Disruption of OPC migration and proliferation under hypoxia stress.*

Additional Figure 3 Disruption of OPC migration and proliferation under hypoxia stress.
(A) Experimental timeline diagram of the time-lapse imaging of OPC migration. Created with Adobe Photoshop (Version 20.0.0). (B) Representative longitudinal fluorescent images of OPCs in the dSC of control and CoCl_2_-treated zebrafish larvae, tagged with GFP under the Olig2 promoter, across various time points (51–63 hpf). CoCl_2_ treatment reduced dorsal migrating OPC numbers and OPC-migration velocity. Scale bar: 50 μm. (C) Quantification of the dorsal migrating OPCs number at different time points, with a statistically significant difference between control and CoCl_2_-treated larvae (*P* = 0.2084 (51 hpf), 0.0052 (53 hpf), 0.0124 (55 hpf), 0.0006 (57 hpf), 0.0008 (59 hpf), 0.0013 (61 hpf), 0.0003 (63 hpf), *n* = 10 larvae). (D) Velocity of dorsal OPC migration. Bar graph shows the mean velocity of OPC migration, with significant reduction in the CoCl_2_-treated group compared to controls (*P* = 0.019, *n* = 10 larvae). (E) OPC proliferation rate. The number of proliferating OPCs, which divided from one into two daughter OPCs during the time-lapse imaging, is significantly reduced in the CoCl_2_ group (*P* = 0.0001, *n* = 10 larvae). Two-way analysis of variance followed by Bonferroni *post hoc* test (C) and two-tailed unpaired *t*-test (D and E) were used. ^*^P < 0.05, ^**^P < 0.01, ^***^P < 0.001, ^****^P < 0.0001; error bars indicate mean ± SEM. CoCl_2_: Cobalt chloride; hpf: hours post-fertilization; Olig2: oligodendrocyte transcription factor 2; OPC: oligodendrocyte progenitor cell.

***Additional Figure 4:*** *Hypomyelination of OLs is mediated by CoCl_2_-induced hypoxia-like state.*

Additional Figure 4 Hypomyelination of OLs is mediated by CoCl_2_-induced hypoxia-like state.
(A) Representative imaging of OL populations (9–12 somites). Longitudinal fluorescent images display the MBP+ mature OLs along the anterior-to-poster and dorsal-to-ventral axis of the SC in control and CoCl_2_-treated larvae. Scale bar: 40 μm. (B) Quantification of dorsal mature OL number (*P* < 0.0001 (3 dpf), < 0.0001 (4 dpf), < 0.0001 (5 dpf), *n* = 10 larvae). (C) Quantification of ventral mature OL number. Bar graph depicts the number of mature OLs in the vSC (*P* = 0.009 (3 dpf), < 0.0001 (4 dpf), < 0.0001 (5 dpf), *n* = 10 larvae). (D) Fluorescence imaging of myelination (9–12 somites). Longitudinal fluorescent images of zebrafish SC illustrate myelination levels. Scale bar: 40 μm. (E) Quantification of dorsal myelination. MBP+ intensity (*P* = 0.003 (3 dpf), < 0.0001 (4 dpf), 0.0004 (5 dpf), *n* = 10 larvae) in the dSC, demonstrating a significant reduction in the CoCl_2_ group across all time points. (F) Quantification of ventral myelination. MBP+ intensity (*P* = 0.003 (3 dpf), < 0.0001 (4 dpf), 0.002 larvae (5 dpf), *n* = 10 larvae) in the vSC are represented in bar graphs, revealing a significant reduction in in the CoCl_2_-treated group compared to controls. (G) Quantification of MBP+ area (*P* < 0.0001 (3 dpf), < 0.0001 (4 dpf), < 0.0001 (5 dpf), *n* = 10 larvae) in the dSC. (F) Quantification of MBP+ area (*P* = 0.0063 (3 dpf), 0.141 (4 dpf), 0.0099 (5 dpf), *n* = 10 larvae) in the vSC. Two-way analysis of variance followed by Bonferroni *post hoc* test (B, C, and E–H) was used. ^*^P < 0.05, ^**^P < 0.01, ^***^P < 0.001, ^****^P < 0.0001; error bars indicate mean ± SEM. CoCl_2_: Cobalt chloride; dpf: days post-fertilization; MBP: myelin basic protein; OL: oligodendrocyte; SC: spinal cord.

***Additional Figure 5:*** *Hypomyelination of OLs under low oxygen concentration-induced hypoxia state.*

Additional Figure 5 Hypomyelination of OLs under low oxygen concentration-induced hypoxia state.
(A) Fluorescence imaging of myelination (9–12 somites). Longitudinal fluorescent images of zebrafish SC illustrate myelination levels. Scale bar: 40 μm. (B, C) Quantification of dorsal myelination. MBP+ intensity and area in the dSC, demonstrating a significant reduction in the hypoxia group across all time points. (D, E) Quantification of ventral myelination. MBP+ intensity and area in the vSC are represented in bar graphs, revealing a significant reduction in hypoxia group compared to controls. Two-way analysis of variance followed by Bonferroni *post hoc* test (B–E) was used. ^*^P < 0.05, ^**^P < 0.01, ^***^P < 0.001, ^****^P < 0.0001; error bars indicate mean ± SEM. CoCl_2_: Cobalt chloride; dpf: days postfertilization; MBP: myelin basic protein; SC: spinal cord.

***Additional Figure 6:*** *Impairment of dynamic myelination by individual OL under CoCl_2_-induced hypoxia stress.*

Additional Figure 6 Impairment of dynamic myelination by individual OL under CoCl_2_-induced hypoxia stress.
(A) Diagram of single OL labeling by Sox10:eGFP-CAAX microinjection into fertilized eggs at one-cell stage. Timeline indicating the dechorionation of zebrafish embryos at 1 dpf, followed by the initiation of CoCl_2_ treatment at 1 dpf, with imaging procedures taking place at 3–5 dpf. Created with Adobe Photoshop (Version 20.0.0). (B) Typical case of single OLs and the myelin segments labeled by Sox10:eGFP-CAAX from CoCl_2_-treated larvae and control at 5 dpf. CoCl_2_ treatment induced significant reductions in the number and the length of myelin sheaths formed by individual OLs. Scale bar: 40 μm. (C, D) Statistics of the number (*P* = 0.0003, *n* = 10 OLs, one OL was got from one fish) and length (*P* = 0.0068, *n* = 10 OLs, one OL was got from one fish) of myelin sheath segments from an individual OLs from CoCl_2_-treated larvae and control at 5 dpf. (E) Representative longitudinal fluorescent images of OLs from control and CoCl_2_-treated zebrafish larvae, tagged with GFP under the Sox10 promoter, across 3–5 dpf. Time-lapse imaging revealed that CoCl_2_ treatment inhibits process of myelin fragment formation at individual OL developmental stages. Scale bar: 4 μm. (F–H) Quantification of dynamic myelinating procession. Myelin sheath total length (*P* < 0.0001 (3 dpf), = 0.0013 (4 dpf), < 0.0001 (5 dpf), *n* = 10 OLs, one OL was got from one fish) myelin sheath length (*P* = 0.0005 (3 dpf), 0.0059 (4 dpf), 0.0039 (5 dpf), *n* = 10 OLs, one OL was got from one fish) and myelin sheath number (*P* = 0.0033 (3 dpf), 0.0021 (4 dpf), 0.0015 (5 dpf), *n* = 10 OLs, one OL was got from one fish) per OLs across 3–5 dpf, demonstrating significant reductions in CoCl_2_ group across all time points. Two-way analysis of variance followed by Bonferroni *post hoc* test (F–H) and two-tailed unpaired *t*-test (C, D) were used. ^**^P < 0.01, ^***^P < 0.001, ^****^P < 0.0001; error bars indicate mean ± SEM. CoCl_2_: Cobalt chloride; dpf: days post-fertilization; MBP: myelin basic protein; OL: oligodendrocyte; SC: spinal cord; SEM: standard error of mean; Sox10: SRY-box transcription factor 10.

***Additional Figure 7:*** *Hypomyelination and oxidative stress are mediated by CoCl_2_-induced hypoxia in vitro.*

Additional Figure 7 Hypomyelination and oxidative stress are mediated by CoCl_2_-induced hypoxia *in vitro*.
(A) Immunostaining of MBP and Hoechst in the primary OLs in control and CoCl_2_-treatment conditions. CoCl_2_ treatment inhibited rat OPC differentiation. Scale bar: 100 μm. (B) Quantification of percentage of MBP^+^ cells. (C) Immunostaining of DCFH-DA-FITC in the primary OLs in control and CoCl_2_-treatment conditions. CoCl_2_ treatment increased intracellular ROS levels of OLs. Scale bar: 100 μm. (D) Quantification of DCFH-DA-FITC intensity. (E) Immunostaining of JC-1 aggregates and monomers in the primary OLs in control and CoCl_2_-treatment conditions. CoCl_2_ treatment induced mitochondrial dysfunction of OLs. Scale bar: 100 μm. (F) Quantification of percentage of JC-1 monomers/aggregates fluorescence. Two-tailed unpaired *t*-test (B, D, F) was used. ^***^P < 0.001, ^****^P < 0.0001; error bars indicate mean ± SEM. CoCl_2_: Cobalt chloride; MBP: myelin basic protein; OLs: oligodendrocytes: OPC: oligodendrocyte progenitor cell; ROS: reactive oxygen species.

## Data Availability

*All data relevant to the study are included in the article or uploaded as additional files.*
